# The versatility of macrophage heterogeneity in liver fibrosis

**DOI:** 10.3389/fimmu.2022.968879

**Published:** 2022-08-05

**Authors:** Chun-Chen Gao, Jian Bai, Hua Han, Hong-Yan Qin

**Affiliations:** ^1^ State Key Laboratory of Cancer Biology, Department of Medical Genetics and Developmental Biology, Fourth Military Medical University, Xi’an, China; ^2^ State Key Laboratory of Cancer Biology, Department of Biochemistry and Molecular Biology, Fourth Military Medical University, Xi’an, China

**Keywords:** liver fibrosis, kupffer cells (KCs), monocyte-derived macrophages, notch signaling, hepatic stellate cells

## Abstract

Liver fibrosis is a highly conserved wound healing response to liver injury, characterized by excessive deposition of extracellular matrix (ECM) in the liver which might lead to loss of normal functions. In most cases, many types of insult could damage hepatic parenchymal cells like hepatocytes and/or cholangiocytes, and persistent injury might lead to initiation of fibrosis. This process is accompanied by amplified inflammatory responses, with immune cells especially macrophages recruited to the site of injury and activated, in order to orchestrate the process of wound healing and tissue repair. In the liver, both resident macrophages and recruited macrophages could activate interstitial cells which are responsible for ECM synthesis by producing a variety of cytokines and chemokines, modulate local microenvironment, and participate in the regulation of fibrosis. In this review, we will focus on the main pathological characteristics of liver fibrosis, as well as the heterogeneity on origin, polarization and functions of hepatic macrophages in the setting of liver fibrosis and their underlying mechanisms, which opens new perspectives for the treatment of liver fibrosis.

## Introduction

Liver fibrosis is a coordinated protective response to acute and/or chronic injury of the liver. A series of cellular and molecular responses could cause pathological changes including death of parenchymal cells and deposition of ECM in the liver (1). As is known that, infection with viruses or parasites, excessive alcohol, nonalcoholic fatty liver, toxins, biliary obstruction, autoimmune disorders and metabolic diseases are the leading causes of liver fibrosis (2, 3). Besides, genetic mutations may also be the cause of liver fibrosis (4). For example, mutations in patatin-like phospholipase domain containing protein 3 (PNPLA3) are closely related to fibrosis caused by alcoholic liver injury or fatty liver (5). Hepatitis C virus-induced liver fibrosis is also associated with a series of genetic mutations (6).

Different types of stimuli mentioned above could cause destruction to the liver and induce a series of repair processes. The very first intention of these repair processes is to maintain normal functions of the liver and resist the damage of harmful stimuli. However, when these harmful stimuli persist, the repair processes tend to lose balance and aggravate the destruction of the structure and normal functions, which could lead to liver fibrosis. Fibrosis is usually caused by destruction of epithelial cells and even some types of endothelial cells that die in the forms of necrosis, apoptosis, programmed necrosis, pyroptosis, ferroptosis, etc. (7). In the meanwhile, tissue resident macrophages could be activated after the recognition of damage related molecular patterns (DAMPs) released by damaged cells through pattern recognition receptors (PRRs), and then release large amount of inflammation-related factors such as cytokines and chemokines (8, 9). These chemokines could further recruit a large number of immune cells, including lymphocytes, polymorphonuclear leukocytes, eosinophils, basophils, mast cells and macrophages to the site of injury, to take part in inflammatory responses (4). In addition, small molecules generated by injury release into the blood, which further attract phagocytic cells, promote their phagocytosis abilities to fulfill the removal of cellular debris and harmful substances in the tissue caused by injury (10). However, in many cases, the above mechanisms may not be able to completely remove harmful substances when injury continues to persist, and as a result, inflammatory response will be amplified and injury will last longer. In this process, activated immune cells could promote the activation of quiescent effector cells by releasing alarmins, cytokines and chemokines on the one hand. And on the other hand, reactive oxygen species (ROS), lipid, acetaldehyde, as well as inflammatory mediators secreted by immune cells such as interleukin-1 β (IL-1β), IL-6, IL-13, IL-33 and tumor necrosis factor α (TNF-α) could further aggravate the death of hepatocytes and cholangiocytes, leading to the destruction of tissue integrity and fibrosis progression (7).

During fibrosis progression, fibroblasts and myofibroblasts are considered as the main effector cells, which play a role in promoting the synthesis of ECM, upregulating pro-inflammatory cytokines, chemokines and angiogenesis related cytokines, while aggravating the impairment of hepatocytes and cholangiocytes (11, 12). In recent years, a series of studies have revealed the origins of fibrotic effector cells, which include tissue resident fibroblasts, bone marrow-derived circulating fibroblasts, vascular smooth muscle cells, and perivascular gli1^+^ mesenchymal stem cell like cells. Besides, epithelial and endothelial cells could also obtain the phenotype of myofibroblasts through activation, transformation, proliferation, infiltration, epithelial-mesenchymal transformation, mesenchymal transformation and endothelial-mesenchymal transformation (13). In addition, mesenchymal cells like hepatic stellate cells (HSCs) and other resident mesenchymal stem cells or precursor cells also appear to be precursors of myofibroblasts, which contribute to the progression of fibrosis (14).

ECM synthesized by fibroblasts and myofibroblasts during fibrosis is the main component of fibrous scar, mainly including type I and type III collagen, fibronectin, elastin, basement membrane proteins such as laminin, and a small number of other kind of proteins, among which type I collagen is the most abundant protein in fibrotic tissue. Fibroblasts and myofibroblasts first secrete procollagen into the tissue, and mature collagen fibers are then formed through modification, shearing and cross-linking (14). In addition, contractile myofibroblasts could synthesize large amount of smooth muscle protein, such as α-smooth muscle actin (α-SMA). The contraction ability of these cells could lead to the twist of normal parenchymal structures, which promotes fibrosis progression and aggravates liver failure (15). And when chronic stimuli persist, fibrotic effector cells like fibroblasts and myofibroblasts appear to be in a state of continuous activation, and fibrous scars further accumulate in the injured tissue, which will worsen tissue impairment.

In the progression of fibrosis, many types of molecular signaling pathways are involved. It is reported that both immune cells and fibrotic effector cells can synthesize and release transforming growth factor-beta (TGF-β) into the tissue in autocrine or paracrine manners. As an essential fibrogenic factor, TGF-β could promote a large number of fibrotic effector cells to synthesize ECM on the one hand, and on the other hand, TGF-β is also an important regulatory factor which could inhibit excessive inflammatory responses (16). Apart from TGF-β, platelet-derived growth factor (PDGF), connective-tissue growth factor (CTGF) and vasoactive peptide system including angiotensin II and endothelin I also contribute to the progression of fibrosis (17). As a potential mitogen and chemokine in the liver, PDGF could promote the proliferation and recruitment of myofibroblasts (18, 19). In the vasoactive peptide system, endothelin also participates in the progression of fibrosis, mainly through G protein coupled endothelin A or endothelin B receptors (20). In addition, angiogenic signaling pathways and integrins may also participate in the regulation of fibrosis. For example, integrins can promote proliferation, migration, differentiation, survival and apoptosis of myofibroblasts. And in the progression of liver fibrosis, αv integrins are upregulated in myofibroblasts (21). Recent studies have shown that stiffness of the tissue is also an important factor for the maintenance of myofibroblasts activation, depending on stress-dependent activation of TGF-β signaling (22).

However, fibrosis is reversible in most cases. Even when fibrosis develops to late stages, it is not a unidirectional irreversible process. When the stimuli of liver injury are removed, the reparative mechanisms start, which inhibit the activation of myofibroblasts. In the meanwhile, local microenvironment is shifted from a pro-inflammatory state to a reparative state, with immune cell, especially macrophages switching from a pro-inflammatory state to a reparative one. During the resolution phases, myofibroblasts in the liver undergo apoptosis, senescence, or inactivation, which is key to fibrosis regression. Therefore, excessive ECM is degraded by matrix metalloproteinases (MMPs), and macrophages contribute to the reversal of fibrosis by phagocytizing ECM fragments and inhibiting the expression of tissue inhibitors of MMPs (TIMPs) (23, 24).

Hepatic macrophages play a central role in the pathogenesis of chronic liver injury and are considered as potential targets for anti-fibrosis treatment. However, through experimental liver fibrosis models, researchers found that hepatic macrophages actually play dual roles by both promoting and eliminating the excessive deposition of ECM (17). In recent years, researchers continue to focus on elaborating the mechanisms of the diverse functions of hepatic macrophages in the process of liver fibrosis, and have found that origins of macrophage subsets, their differentiation and their polarization states might be the reasons why they function differently during fibrosis. In this review, we will summarize current knowledge on the heterogeneity of hepatic macrophages in the progression and resolution of fibrosis. In-depth understanding of heterogeneity and various functions of hepatic macrophages will open new perspectives for macrophage-based interventional strategies in the treatment of liver fibrosis.

## Heterogeneity of hepatic macrophages

### Origins of hepatic macrophages

Hepatic macrophages are abundant in the liver, which account for 80% of the total macrophages in the body (25). Macrophages are important contributors in the maintenance of homeostasis of the liver, and could sense integrity of liver by identifying and removing bacteria and microbial debris obtained from small intestine through portal vein, in order to determine the initiation or inhibition of immune response (8, 26). According to previous concepts, KCs generally referred to all kinds of macrophages in the liver, and they were roughly identified by surface markers like F4/80 (specifically expressed in mouse) or CD68 (mainly expressed in human). However, according to recent findings, hepatic macrophages show strong heterogeneity after liver injury, and can be divided into embryonic tissue resident macrophages (KCs) and monocyte derived macrophages based on their origins. The different origins of hepatic macrophages are closely related to their functional diversity during fibrosis (27).

KCs are the first line of defense against microbial invasion and maintain homeostasis of the liver, which preferentially reside in periportal and mid zones of murine liver and locate in the mid zones of human liver, usually with larger size than monocyte derived macrophages (28, 29). KCs function as main phagocytic macrophages which clear exogenous pathogens, engulf aging red blood cells and participate in the regulation of iron metabolism and lipid metabolism (30). However, due to continuous exposure of the liver to intestinal antigens and low-dose bacterial endotoxin, KCs avoid excessive self-activation through a variety of mechanisms. For example, KCs maintain immune tolerance through secreting anti-inflammatory cytokines like IL-10 and modulating regulatory T cells (Tregs) (27). Murine KCs were previously recognized as CD45^+^F4/80^+^CD11b^int^CLEC4F^+^TIMD4^+^ cells, and additional surface markers including V-set and immunoglobulin domain containing 4 (VSIG4) and folate receptor beta (FOLR2) were identified to better characterize KCs. And as for human KCs that were generally characterized as CD68^+^ TIMD4^+^ cells, VSIG4 was found to be one of the best human KC markers according to cellular indexing of transcriptomes and epitomes by sequencing (CITE-seq) data, while CD5L, FOLR2, CD163, and CD169 were also useful markers to identify human KCs (28).

KCs are tissue resident macrophages and mainly derived from primitive hematopoiesis of yolk sac and the definitive hematopoiesis of fetal liver. According to recent researches, embryonic hematopoietic stem cells could also be progenitors of hepatic macrophages. Monocytes unlikely contribute to adult macrophages pool in steady state, and KCs usually maintain through self-renewal (31). However, with single cell sequencing technology, recent researches indicated that there appear to be two subsets of KCs with distinct functions in human liver, one of which support tolerogenic immune responses, while the other show pro-inflammatory phenotype (32). And in murine liver, a major CD206^lo^ESAM^-^ subset (KC1) and a minor CD206^hi^ESAM^+^ subset (KC2) were also identified. KC2 exhibit a distinct metabolic signature, which regulate oxidative stress associated with obesity. And this minor subset of KCs are equipped with enriched IL-2 sensing machinery and antigen presentation capacity (33, 34). In addition, a subset of radioresistant KCs were discovered, which highly express cyclin dependent kinase inhibitor 1A (CDKN1a) (35). During mouse and human nonalcoholic steatohepatitis (NASH) pathogenesis, a specific Trem2^+^ NASH-associated KC population was identified (36). These new findings have challenged our previous concept. However, the above results still require further analysis to confirm whether these subsets of KCs truly come from different origins, and whether pro-inflammatory KCs are actually monocyte derived macrophages.

In homeostatic conditions, there are only a few monocytes derived macrophages in the murine liver, which originate from CX3CR1^+^CD117^+^Lin^-^ bone marrow derived progenitors and modulate immune responses (37). Murine monocytes can be further divided into different subsets by lymphocyte antigen 6 complex, locus C (Ly6C). Ly6C^hi^ monocytes are also CCR2^hi^CX3CR1^lo^CD62L^+^ cells, which are rapidly recruited to the site of injury and differentiate into monocyte derived macrophages when tissue damage occurs. In comparison, Ly6C^lo^ monocytes are CCR2^lo^CX3CR1^hi^ cells, showing a patrolling behavior in the liver and expressing more scavenging receptors (27). Based on CITE-seq data, other non-KC macrophage subsets were identified, namely GPNMB^+^SPP1^+^ lipid-associated macrophages (LAMs), GPNMB^+^ bile duct LAMs, CD207^+^CX3CR1^+^ liver capsular macrophages (LCMs), as well as transitioning monocytes (28, 38, 39). Different subsets of non-KC macrophages seem to be located in different zones of the liver, for example, LCMs occupy the hepatic capsule, which function in neutrophil recruitment in response to bacteria reaching the liver capsule (39). Besides, it is reported that LAMs were found only in portal zones of non-steatotic livers, while in steatosis liver, LAMs were located across portal, periportal and mid zones (28). The above findings further suggest the need for spatial approaches to better reveal cell identities. Different from murine monocytes, human monocytes could be divided by CD14 and CD16 (40). Beside bone marrow, peritoneum and spleen are also resources of hepatic macrophages (41, 42).

With different origins and locations in the liver, identities and functions of hepatic macrophage subsets are shaped by both ontogenic and environmental factors, which could possibly account for their diverse functions in response to different conditions of the liver. It is interesting that monocytes colonizing the liver macrophages niche could be imprinted with KC identity. Interactions of the delta like canonical Notch ligand 4 (DLL4) and TGF-β family ligands produced by endothelial cells, as well as liver X receptor alpha (LXR-α) induced by endothelial and stellate cells are required for the fate of macrophages migrating to the liver followed by the maintenance of KC identity. Therefore, signals from the tissue microenvironment could shape the identity of macrophages migrating to the liver to acquire tissue-specific phenotypes (43, 44). However, underlying epigenetic mechanisms maintain the status of these migrated macrophages in liver still need to be further investigated.

### Polarization of hepatic macrophages

Different activation states of hepatic macrophages are also closely related to their functional diversity during fibrosis. According to the old dogma, hepatic macrophages was described as M1 and M2 macrophages. M1 macrophages are defined as classically activated macrophages under the stimulation of interferon-γ (IFNγ) or lipopolysaccharide (LPS). M1 macrophages take part in promoting inflammation and Th1 immune response, and exert anti-fibrotic role. In comparison, M2 macrophages are alternatively activated macrophages under the stimulation of IL-4 or IL-13. M2 macrophages are anti-inflammatory cells promoting Th2 immune response as well as tissue repair and regeneration (8, 45).

However, in recent years, with the advent of single cell sequencing technology, researchers have revealed that even under physiological conditions, there are various macrophage subsets with different activation states and diverse functions in the liver. Stimulated by complex signals in microenvironment, these macrophages actually show a broad spectrum of activation states instead of a well-defined M1 or M2 phenotype. In injured liver, macrophages usually express both inflammation and resolution markers, and could change their phenotypes under different microenvironments. Therefore, instead of classical M1/M2 dichotomy, definition of activators and a collection of markers that describe activation states of the macrophages should be utilized, such as M(IL-4), M(IL-10) and M(TGF-β) and so on (46). These findings may possibly explain why hepatic macrophages play different or even completely opposite roles in different stages of liver fibrosis.

### Functions of hepatic macrophages in liver fibrosis

Evidence from clinical and animal studies has shown that hepatic macrophages play important roles in the process of liver fibrosis. Upon injury, hepatic epithelial cells such as hepatocytes or cholangiocytes are destroyed, which leads to the release of DAMPs like high mobility group box 1 (HMGB1) (17, 47). Interaction of HMGB1 with its receptor then triggers signal transduction cascades, which could possibly result in cellular responses like inflammation and fibrosis (48). Researches have indicated that liver fibrosis caused by multiple etiologies might yield context-dependent functions of different hepatic macrophage subsets. For example, in alcohol-related cirrhosis, hepatic macrophages express both M1 and M2 macrophage-associated cytokines, and are more sensitive to endotoxin like LPS. Moreover, ethanol could enhance the expression of telomerase reverse transcriptase, which tends to promote M1 hepatic macrophage polarization. While in liver fibrosis caused by Hepatitis B virus (HBV) and Hepatitis C virus (HCV) infections, hepatic macrophages have a significant immunoregulatory function and appear to play a role in pathogen clearance and anti-viral immunity. Evidence suggests that TNFα released by hepatic macrophages could induce HCV entry of hepatoma cells. However, other cytokines such as IL-1β and IL-6 could inhibit the replication of HCV, indicating that hepatic macrophages play diverse roles in the context of HCV infection. And in fibrosis induced by NAFLD, hepatic macrophages accumulate dramatically, which show a M2 macrophage phenotype at the early stage and a M1 macrophage at the late stage (45, 49). Despite exhibiting different functions in response to diverse stimuli, hepatic macrophages are inclined to play similar roles in the progression and resolution of liver fibrosis, which are discussed as follows.

#### A. Kupffer cells

Upon injury, KCs are activated and initiate immune responses through rapidly secreting cytokines and chemokines, such as IL-1β, TNF-α, CCL2 and CCL5, and then recruit other types of immune cells such as monocytes to infiltrate the site of injury (8, 25). However, the number of KCs decline rapidly in the initiation stage of fibrosis, and gradually recover with the regression of inflammation and resolution of fibrosis. Besides, KCs could secrete pro-fibrotic cytokines like TGF-β and PDGF to activate HSCs, which aggravate the progression of fibrosis. On the other hands, KCs could also express many types of MMPs such as MMP9, MMP12 and MMP13 to promote degradation of ECM and contribute to the resolution of fibrosis (50, 51).

#### B. Monocyte-derived Ly6C^hi^ macrophages (Ly6C^hi^Ms)

By contrast, monocyte-derived macrophages are significantly accumulated after liver injury. In CCl_4_ induced liver fibrosis models, the number of macrophages in the liver amplified 3-5 times due to the recruitment of Ly6C^hi^ monocytes. After recruited to the liver, Ly6C^hi^ monocytes differentiate to Ly6C^hi^ macrophages which secrete inflammatory cytokines like TNF, IL-6 and IL-1β, as well as chemokines like CCL2, CCL3 and CCL5 to promote the recruitment of other leukocytes (52). Although these monocyte-derived Ly6C^hi^ macrophages initially showed a pro-inflammatory and pro-fibrotic phenotype, they further differentiated into Ly6C^lo^ macrophages with anti-inflammatory functions which promote tissue repair and resolution of fibrosis (53). CCL2-CCR2 pathway is essential for the recruitment of Ly6C^hi^ monocytes (54). Knockout of CCR2 or inhibition of CCL2 could alleviate liver fibrosis, indicating that Ly6C^hi^ monocytes/macrophages are pro-fibrotic cells and play roles in aggravating tissue damage (55, 56).

Excessive deposition of ECM is one of the main pathological features of liver fibrosis. Studies have shown that macrophages can promote the deposition of ECM through a variety of mechanisms resulting in accelerated progression of fibrosis. Ly6C^hi^ macrophages generate cytokines like TGF-β, PDGF, CTGF and IL-13 to activate HSCs and other interstitial precursors. TGF-β is one of the main contributors to ECM synthesis in the tissue, while upregulating the expression of α-SMA in activated myofibroblasts and generating type I collagen (16). PDGF acts as a mediator to promote the proliferation of activated myofibroblasts in the process of fibrosis through extracellular signal regulated kinase (ERK)-dependent or independent manners. Meanwhile, PDGF, IL-4 and IL-13 secreted by Ly6C^hi^ macrophages could directly enhance the synthesis of ECM by myofibroblasts (20). In addition, macrophages could also express chemokines like CCL8 and CCL7 to further recruit myofibroblasts to the site of injury. It is reported that Galectin3 secreted by macrophages could promote the activation of myofibroblasts in liver fibrosis models (57). Recent studies have indicated that pro-inflammatory cytokines like TNF and IL-1β secreted by macrophages could also activate HSCs, and maintain the survival of activated HSCs through nuclear factors κB (NF-κB) signaling pathway, which aggravates liver fibrosis (58). In addition, there appear to be other factors that participate in the regulation of macrophages during liver fibrosis progression. For example, alcohol could increase intestinal permeability, thus the level of LPS in the circulation is enhanced and activates HSCs and KCs in liver through TLR4 signaling pathway, which lead to the progression of liver fibrosis (59).

#### C. Monocyte derived Ly6C^lo^ macrophages (Ly6C^lo^Ms)

However, hepatic macrophages function differently in the process of fibrosis, with both pro-fibrotic and anti-fibrotic functions, which may be due to the opposite roles of different macrophage subsets in the tissue. Under pathological microenvironment, Ly6C^hi^ macrophages switch their phenotype towards Ly6C^lo^ macrophages triggered by specific molecular signals, which is an indicator of fibrosis resolution. It is reported that factors like phagocytosis of cellular debris could promote the phenotypic switch of these macrophages. According to in-depth gene expression profiling, Ly6C^lo^ macrophages are the main sources of MMPs such as MMP9, MMP12 and MMP13, which accelerate the resolution of ECM. Ly6C^lo^ macrophages express high levels of TNF-related apoptosis-inducing ligand (TRAIL), which could promote myofibroblast apoptosis together with MMP9. Clearance of Ly6C^lo^ macrophages hinders the resolution of liver fibrosis (50). In addition, when CCL2-CCR2 signaling pathway is blocked in CCl_4_ induced or methionine choline deficiency diet induced mouse models, the number of Ly6C^lo^ macrophages rises dramatically, leading to the rapid resolution of liver fibrosis (56). Interestingly, Ly6C^lo^ macrophages express low levels of pro-inflammatory cytokines and chemokines, while their expression of anti-inflammatory cytokines such as CX3CR1, IL-10 and arginase 1 is enhanced (60, 61). The above findings further indicate that Ly6C^lo^ macrophages are the main contributors to fibrosis resolution and tissue repair. It is worth noticing that pro-inflammatory Ly6C^hi^ macrophages and reparative Ly6C^lo^ macrophages could express markers of both M1 and M2 macrophages, which indicates that M1/M2 dichotomy is not adequate to explain the heterogeneity of hepatic macrophages in the context of liver fibrosis (27).

Taken together, different functions of hepatic macrophage subsets during fibrosis might be attributed to their heterogeneity on origins, locations and activation. A table summarizing the heterogeneity of hepatic macrophages is provided (**Table 1**).

**Table 1:** Heterogeneity of hepatic macrophages.

	Kupffer cells	Monocyte derived macrophages
**Origins**	Derived from yolk sac, fetal liver and embryonic hematopoietic stem cells	Originated from CX3CR1^+^CD117^+^Lin^-^ bone marrow derived progenitors
**Features**	Tissue resident, self-renewal	Circulating, with a half-life of 2 days or 20 hours
**Locations**	Periportal and mid zones (mouse); Mid zones (human)	Portal zones (Ly6C^hi^/Ly6C^lo^Ms, healthy LAMs); Hepatic capsule (LCMs); Portal, periportal and mid zones (steatosis LAMs)
**Morphologies**	Stellate	Circular
**Markers**	**Mouse:** CD45^+^F4/80^+^CD11b^int^CLEC4F^+^TIMD4^+^ VSIG4^+^FOLR2^+^STAB2^+^; **Human:** CD68^+^TIMD4^+^VSIG4^+^ FOLR2^+^CD163^+^CD169^+^	**Mouse:** CCR2^hi^CX3CR1^lo^CD62L^+^ or CCR2^lo^CX3CR1^hi^ CD11b^hi^F4/80^int-lo^; **Human:** CX3CR1^lo^CD14^+^CD11b^hi^CD11c^+^CD62L^+^CD16^–^ or CX3CR1^hi^CD14^lo^CD16^+^CD11b^+^CD11c^hi^
**Subsets**	**KC1:** CD206^lo^ESAM^-^ (major); **KC2:** CD206^hi^ESAM^+^ (minor); **Radioresistant KCs:** CDKN1a^hi^; **NASH-associated KCs:** Trem2^+^	**Ly6C^hi^Ms:** CCR2^hi^CX3CR1^lo^CD62L^+^; **Ly6C^lo^Ms:** CCR2^lo^CX3CR1^hi^; **LAMs:** GPNMB^+^SPP1^+^; Trem2^+^; **Bile duct LAMs:** GPNMB^+^; **LCMs:** CD207^+^CX3CR1^+^
**Functions**	Main phagocytic macrophages; Regulation of iron and lipid metabolism; Immune tolerance	Major immune response orchestrators; Inflammatory capacity, angiogenic and fibrogenic activity; Immune-suppressive functions

## Hepatic macrophages in humans

Although we have understood the liver fibrogenesis using rodent models, there still remains unmatched conditions in fibrotic patients. Previously, a subset of CD14^lo^CD16^-^ tissue resident KCs were found in the liver of cirrhosis patients, while CD14^hi^CD16^-^ and CD14^+^CD16^+^ macrophages are defined as monocyte derived macrophages in humans. And CD14^+^CD16^+^ macrophages are the most abundant in cirrhosis livers. It is reported that CD14^+^CD16^+^ macrophages could be derived from CD14^hi^CD16^-^ macrophages, which resembles the phenotypic switch from Ly6C^hi^ macrophages to Ly6C^lo^ macrophages in mice. Although CD14^+^CD16^+^ macrophages show phagocytic and reparative capacities, they could also express pro-inflammatory and pro-fibrotic cytokines to directly active HSCs, which resembles the features of Ly6C^hi^ macrophages to some degree (62).

Currently, single-cell RNA sequencing (scRNA-seq) is being applied in exploring the mechanisms regulating human liver fibrosis. Ramachandran and colleagues take advantage of scRNA-seq and find that a novel scar-associated Trem2^+^CD9^+^ macrophage subsets (SAMs) existing in human fibrotic liver, which differentiates from circulating monocytes and exhibits pro-fibrogenic phenotype. In addition, there are tissue resident KCs and monocytes in the fibrotic niche, but no differentiation from KCs to SAMs and no progression from SAMs to KCs. More importantly, the SAM subsets are conserved across species, suggesting Trem2^+^CD9^+^ SAMs might be a potential pathology biomarker related with hepatic fibrogenesis (63). However, how differently these hepatic macrophage subsets play roles in human hepatic fibrosis still requires further investigations.

## Regulation of hepatic macrophages in liver fibrosis

Many signaling pathways are involved in the initiation, progression and resolution of liver fibrosis. In chronic liver diseases caused by bacterial infection, pathogen associated molecular patterns (PAMPs) participate in the response of fibrosis by activating TLR. By applying TLR4 antibodies or TLR4 deficient mice, liver fibrosis is alleviated (64). And in patients with liver fibrosis caused by HCV, inhibition of TLR4 is also related to the alleviation of fibrosis (6). In mouse models of liver fibrosis induced by alcoholic liver disease or nonalcoholic fatty liver disease, TLR4 signaling mainly promotes the production of proinflammatory cytokines by KCs, including TNFα, IL-1β, CCL2 and CCL20 (65, 66). In addition, TLR9 expressed on HSCs can also be activated by DNA fragments released from hepatocytes, which aggravates the progression of liver fibrosis, while TLR3 and TLR7 signaling could impede liver fibrosis progression (59).

Researches have shown that peroxisome proliferator-activated receptors (PPARs) are essential regulators of inflammation in macrophages and are involved in the regulation of liver fibrosis. It was reported that deletion of PPAR-α could worsen hepatic steatosis, while PPAR-α agonist was associated with reversion of NASH and fibrosis. Compared with healthy controls, the level of PPAR-γ in the peripheral blood of HBV patients was significantly decreased. Further studies have found that the transcription of PPAR-γ in HBV patients was inhibited, and might possibly be related to DNA methylation (67). Additionally, the transcriptional inhibition of PPAR-γ is essential for the activation of HSCs, which relies on the binding of MeCP2 to the CpG island in the promoter of PPAR-γ (68). Both PPAR-γ and PPAR-δ contribute to the anti-inflammatory polarization of hepatic macrophages, and deletion of either PPAR isoforms in macrophages could exacerbate hepatic steatosis and fibrosis. Therefore, agonists of PPARs represent attractive candidates for the treatment of hepatic fibrosis, and inhibition of PPARs is closely related to sustained inflammatory state and delayed resolution of fibrosis (69).

Moreover, interferons also participate in the regulation of macrophages in liver fibrosis. Interferons are up-regulated in chronic liver diseases, and are central participators in innate immune responses (64). In various types of immune cells, activation of TLR can promote the secretion of interferons. IFN-γ could induce proinflammatory activation of hepatic macrophages, and controlling the activation of macrophages through interfering with IFN-γ provides a possible therapeutic target against hepatic fibrosis (70). However, it is reported that IFN-γ could hinder the progression of liver fibrosis by inhibiting the proliferation of HSCs and the expression of α-SMA, and by promoting the activation of NK cells (71). And in human liver, IFN-λ is closely associated with antiviral responses and could promote inflammation and fibrosis through stimulating macrophage phagocytosis and the secretion of pro-inflammatory cytokines as well as chemokines by macrophages (72). Besides, IFNα could also contribute to the regulation of fibrosis by downregulating the transcription of collagen synthesis related genes in HSCs (73). Accordingly, interferons could be utilized as therapeutic targets in the treatment of liver fibrosis.

C-Jun-N-terminal kinases (JNKs) could be activated by a variety of stimuli, including TLRs, IL-1β, TNF, ROS and other saturated free fatty acids. The liver could usually express JNK1 and JNK2, instead of JNK3. JNK participates in multiple signaling cascades with relevance to hepatocellular injury, metabolism, inflammation and fibrosis (74). Activation of JNK could promote the release of pro-inflammatory cytokines, attract macrophages to the injured liver and further augment liver fibrosis (75). In addition, in HSCs, JNK plays a role in promoting the progression of fibrosis by enhancing the proliferation and activation of HSCs induced by PDGF, TGF-β and angiotensin II, which contributes to the deposition of ECM (76). Apart from directly aggravating liver fibrosis, JNK also participates in the regulation of liver fibrosis by regulating liver steatosis, death of hepatocytes, and the expression of inflammatory factors (74).

In addition, NF-κB signaling pathway is key to the regulation of cellular processes like inflammation and cell death, thus plays an important role in chronic liver diseases. NF-κB signaling pathway could be activated by a variety of stimuli, including TLRs, IL-1β and TNFα (45). Conditional inhibition of NF-κB in KCs could alleviate the degree of liver fibrosis induced by CCl_4_ (77). When NF-κB signaling pathway is activated in HSCs, the survival of HSCs is prolonged, leading to the sustained fibrosis. Moreover, it is reported that NF-κB signaling in HSCs is up-regulated by the stimulation of IL-1β and TNFα secreted by KCs (58).

JAK-STAT signaling pathway may also be involved in the regulation of liver fibrosis. JAK tyrosine kinase plays a key role in the apoptosis of macrophages. The activation of JAKs leads to the autophosphorylation of JAKs and the phosphorylation of STATs. Studies have shown that the suppressor of cytokine signaling (SOCS) protein could inhibit JAK-STAT signaling pathway, thereby inhibiting the release of inflammatory factors and alleviating the inflammatory responses in the liver (78, 79).

Notch signaling pathway is also an important participator in the regulation of liver fibrosis. We have previously reported that Notch signaling could participate in the regulation of liver fibrosis by regulating the activation of macrophages. When RBP-J gene is conditionally knocked out in myeloid cells, the expression of CYLD is up-regulated, and NF-κB activity and the expression of TGFβ and PDGFβ is inhibited, which alleviated the progression of liver fibrosis (80). In addition, in mouse models of liver fibrosis infected with *Schistosoma japonicum*, inhibition of Notch1/Jagged1 signaling pathway could reverse the M2 polarization of macrophages, thereby alleviating liver fibrosis (81). And in the mouse models of CCl_4_ induced liver fibrosis, the inhibition of Notch signaling could hinder the activation of HSCs, and the polarization of macrophages to a pro-inflammatory M1 phenotype is also inhibited. Meanwhile, the expression of anti-inflammatory genes expression is up-regulated, which contributes to alleviated liver fibrosis (82). In addition, Notch signaling mediates the proliferation of CCR2-independent hepatic macrophages, and thereby regulates the progression of hepatocellular carcinoma (83).

Beside Notch signaling pathway, Wnt/β-Catenin signaling pathway is also involved in the regulation of liver fibrosis. It is reported that conditional blockade of Wnt signaling in myeloid cells could aggravate liver fibrosis, with precursor cells activated, TIMP1 level up-regulated to inhibit collagen degradation, and MMP12 and MMP13 level down-regulated. The above findings suggested that activation of Wnt signaling pathway in macrophages was closely related to the inhibition of liver fibrosis (84, 85).

**Figure 1:**
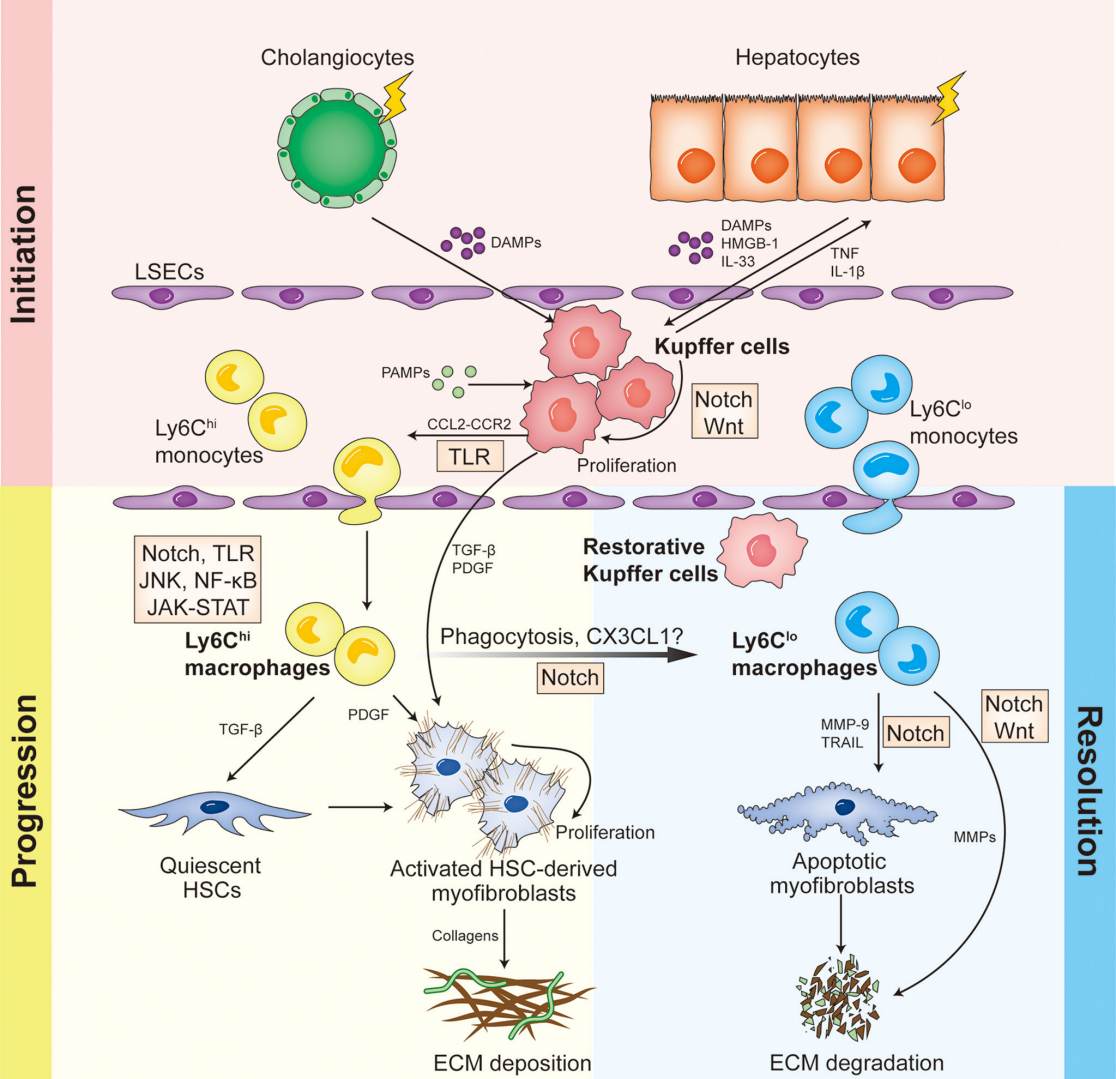
Different hepatic macrophage subsets function differently in distinct stages of liver fibrosis, and are modulated by various signaling pathways and regulatory molecules.

To sum up, different hepatic macrophage subsets function differently in distinct stages of liver fibrosis, and are modulated by various signaling pathways and regulatory molecules (**Figure 1**). Therefore, in-depth study on how macrophages play roles in liver fibrosis and the underlying mechanisms could improve our understanding of the complex regulating network, and open new perspectives for macrophage-based treatment of liver fibrosis.

## Macrophage-directed therapeutic approaches to liver fibrosis

In recent years, hepatic macrophages have already become an attractive target for novel therapeutic approaches to treat liver fibrosis. In humans and mice, the signaling pathways that promote the recruitment and differentiation of macrophages and trigger immune responses are conserved, which should in theory allow the transition from mouse models to human diseases (25, 86). However, targeting macrophages to treat human liver fibrosis faces many challenges. First of all, through animal models, it is found that macrophages play different or even completely opposite roles under different experimental conditions. Therefore, when performing macrophage-directed therapeutic approaches, it is necessary to consider the optimal dosing, the intervention timing and the specific targeted macrophage subsets to treat liver fibrosis in different disease stages. Second, mouse models could not fully represent the conditions of human diseases by far. Mouse models usually could only represent the pathological process of liver fibrosis under specific stimuli, but cannot fully reflect the process of liver fibrosis induced by a variety of different causes in human diseases (86). Moreover, human patients are more heterogeneous than inbred mouse strains, with respect to intrinsic factors like gender, age, genetic background and existing comorbidities as well as external factors like microbiota, infections and combined medication. Third, researchers have a clearer and deeper understanding of the heterogeneity and functions of hepatic macrophages in mouse models than in human diseases, as limitations in obtaining human fibrotic tissues at different stages of diseases hinder the further understanding of hepatic macrophage subsets in humans (87, 88). Despite the above challenges, with advanced technologies researchers have gained in-depth understanding of the role of different hepatic macrophage subsets in the progression and resolution of liver fibrosis, providing theoretical basis for macrophages-targeted treatment.

In the initiation of liver fibrosis, KCs are activated by small molecules generated by injury, which aggravates the pro-inflammatory immune responses in the liver. Therefore, targeting the activation of KCs could be an important intervention for the treatment of liver fibrosis. When broad-spectrum antibiotics were utilized to reduce bacterial translocation and TLR4 dependent macrophage activation, liver fibrosis in mice was significantly reduced (89). Therefore, by using probiotics or antibiotics, transferring fecal microbiota or changing bile acid composition to modify intestinal permeability and microbiome, the activation of KCs could be inhibited and fibrosis could be alleviated. In addition, both hepatic macrophages and hepatocytes are activated by intracellular inflammatory signaling pathways, such as NF-κB, ASK1, JNK and p38 signaling (17). Therefore, inhibitors of specific inflammatory signaling pathways, for example ASK1 inhibitor Selonsertib, not only function on the metabolism of hepatocytes, but also participate in the activation of macrophages. In a multicenter, open-label trial involving 72 patients with nonalcoholic steatohepatitis and liver fibrosis, Selonsertib is found to have significant anti-fibrotic effect (90).

In the progression of liver fibrosis, monocytes are recruited to the site of injury and aggravate inflammatory responses, which is regulated by chemokines and their receptors in mouse models and human diseases, including CCL2-CCR2, CCL1-CCR8, CCL5-CCR1/CCR5, as well as CXCL10-CXCR3 (39). Therefore, a variety of pharmacological strategies targeting chemokines and their receptors have been utilized to interfere with the process of liver fibrosis, including monoclonal antibodies against chemokines or their receptors, chemokine receptor antagonists that prevent chemokine binding, and aptamers or small molecule inhibitors that inhibit chemokines (91). Among various CCR2 inhibitors, the effect of Cenicriviroc, a CCR2/CCR5 co-inhibitor, is verified in the phase II clinical trial of 289 patients with nonalcoholic steatohepatitis and liver fibrosis. Cenicriviroc can effectively prevent the recruitment of monocytes to the site of injury mediated by CCL2, and has an anti-fibrotic effect in mouse models. A randomized controlled trial indicated that the number of patients with liver fibrosis recovered in Cenicriviroc group (20%) is twice as many as that in placebo group (10%), without worsening after one year of the two-year treatment. Moreover, Cenicriviroc trial has excellent safety profile tested by clinical trials, suggesting that inhibition of monocyte recruitment will not affect the antimicrobial defense and immune responses of hepatic macrophages (92, 93).

The above treatment of inhibiting chemokines mainly aims to reduce the recruitment of inflammatory monocytes, thereby alleviating liver fibrosis, while alternative therapeutic interventions focus on augmenting macrophage numbers and functions. When patients with liver fibrosis progress to cirrhosis, the immune responses are seriously damaged with high risks of life-threatening infections (94). Therefore, hematopoietic growth factors that play important roles in the recovery of immune functions are investigated. Researchers found that CSF1-Fc can promote the accumulation of macrophages in the liver of mice, promote the proliferation of KCs, and then facilitate innate immunity in mice after partial hepatectomy or acetaminophen (APAP) induced liver injury, thus delaying the progress of the diseases (95).

In addition, KCs express high levels of scavenger receptors, which can be used for drug delivery. Studies based on mouse models have found that hard-shell microbubbles (size ~2 μm), liposomes (size ~100 nm) and polymers (size ~10 nm) can deliver drugs to the liver, and KCs are the main targets of these drug delivery systems (96). Because KCs express mannose receptor CD206, when particles are functionally modified by sugar moiety mannose, the targeting specificity for KCs will be significantly enhanced (96). Dexamethasone is an anti-inflammatory drug. And with macrophage-targeted delivery of dexamethasone, liver fibrosis in mice was alleviated (97). Therefore, different drug delivery systems can be utilized to deliver specific drugs such as siRNA, inhibitors of inflammatory signaling or enhancers of autophagy to hepatic macrophages, so as to inhibit their pro-fibrotic roles (98). Current studies also suggested that galectin-3 can be used as a target to regulate the function of inflammatory macrophages in advanced liver diseases, and galectin-3 inhibitors are under clinical investigations (99). In addition, as studies have already suggested that the phenotypic switch from pro-inflammatory macrophages to reparative macrophages in the process of liver fibrosis may be regulated by phagocytosis or CX3CR1, identification of the mechanisms of phenotypic switch may provide new translational approaches for clinical interventions of liver fibrosis.

However, the role of macrophage-based therapies remains unclear at present. Theoretically, CD14^+^ monocytes could be extracted from patients with liver cirrhosis by plasma isolation, and these cells could further differentiate into reparative macrophages, suggesting their therapeutic possibilities in clinical trials (100). Although it is reported that transfer of ex-vivo polarized reparative macrophages could alleviate liver fibrosis in mice, not any beneficial effects is observed after transfer of G-CSF mobilized CD133^+^ bone marrow stem cells to patients with cirrhosis (88). Similarly, through the APAP induced or CCl_4_ induced mouse fibrosis model, it is found that transfer of bone marrow derived monocytes could aggravate liver injury and the progression of liver fibrosis (93). These unexpected outcomes might be caused by uncontrolled differentiation fate of precursor cells *in vivo*. Considering this point, Thomas and colleagues find that delivery of unpolarized macrophages can reduce both CCl_4_-induced murine liver fibrosis and human hepatic cirrhosis (101–103). However, due to the high plasticity of macrophages in tissue microenvironement, transplanted unpolaried macrophages may show various phenotypes with different stimuli *in vivo*. We recently transferred the bone marrow-derived unpolarized macrophages and ex-vivo polarized M1 macrophages into mouse fibrosis models respectively. Compared with unpolarized macrophages, we found that M1 macrophages are more qualified to alleviate liver fibrosis through modulating the microenvironment, suggesting that more defined macrophages will enable adoptive cell therapy more precisely for human liver fibrosis in the future (103, 104).

Besides, with genetic programming technologies, genetically modified macrophages have emerged as attractive targets in the treatment of various diseases. By modifying certain transcriptome, macrophages could be reprogrammed to acquire certain therapeutic functions involved in promoting tissue regeneration and wound healing, while inhibiting inflammation, which may potentially contribute to the resolution of liver fibrosis. Genetic modification tools, such as RNA interference knockdown techniques, miRNA transfection and CRISPR/Cas9 gene-editing techniques, appear to be powerful approaches in generating genetically modified macrophages for clinical treatment (105, 106). For example, it is reported that utilizing siRNA against mannose-modified HMGB1 in hepatic macrophages could reduce inflammation and restore liver functions in a NASH mouse model (107). Cell therapy with genetically modified macrophages is a rapidly developing field, which provides a promising target for the treatment of liver fibrosis.

## Conclusions

Hepatic macrophages play central roles in the pathogenesis of chronic liver injury and are considered as potential targets for anti-fibrosis treatment. However, macrophages exert a wide range of different functions during liver fibrosis, which hinders the development of macrophage-directed therapeutic approaches to some extent. Origins of macrophage subsets, their differentiation and their polarization states might be the reasons why they function differently during fibrosis. And in-depth understanding of the mechanisms underlying how macrophages regulate inflammatory responses, wound healing and tissue repair during liver fibrosis will provide new therapeutic strategies for the treatment of fibrotic diseases.

## Author contributions

C-CG and JB wrote the review and designed the figures. HH and H-YQ supervised and edited the manuscript. All authors contributed to the article and approved the submitted version.

## Funding

This work was supported by grants from the National Natural Science Foundation of China (81530018, 31970829, 31730041).

## Conflict of interest

The authors declare that the research was conducted in the absence of any commercial or financial relationships that could be construed as a potential conflict of interest.

## Publisher’s note

All claims expressed in this article are solely those of the authors and do not necessarily represent those of their affiliated organizations, or those of the publisher, the editors and the reviewers. Any product that may be evaluated in this article, or claim that may be made by its manufacturer, is not guaranteed or endorsed by the publisher.

## References

[1] Koyama Y Brenner DA. Liver inflammation and fibrosis. J Clin Invest (2017) 127:55–64. doi: 10.1172/JCI88881 28045404 PMC5199698

[2] Ortiz-Perez A Donnelly B Temple H Tiao G Bansal R Mohanty SK. Innate immunity and pathogenesis of biliary atresia. Front Immunol (2020) 11:329. doi: 10.3389/fimmu.2020.00329 32161597 PMC7052372

[3] Campana L Esser H Huch M Forbes S. Liver regeneration and inflammation: from fundamental science to clinical applications. Nat Rev Mol Cell Biol (2021) 22:608–24. doi: 10.1038/s41580-021-00373-7 34079104

[4] Rockey DC Bell PD Hill JA. Fibrosis–a common pathway to organ injury and failure. N Engl J Med (2015) 372:1138–49. doi: 10.1056/NEJMra1300575 25785971

[5] Tian C Stokowski RP Kershenobich D Ballinger DG Hinds DA. Variant in PNPLA3 is associated with alcoholic liver disease. Nat Genet (2010) 42:21–3. doi: 10.1038/ng.488 19946271

[6] Martinez MA Franco S. Therapy implications of hepatitis c virus genetic diversity. Viruses (2021) 13:41. doi: 10.3390/v13010041 PMC7824680 33383891

[7] Weiskirchen R Weiskirchen S Tacke F. Organ and tissue fibrosis: Molecular signals, cellular mechanisms and translational implications. Mol Aspects Med (2019) 65:2–15. doi: 10.1016/j.mam.2018.06.003 29958900

[8] Krenkel O Tacke F. Liver macrophages in tissue homeostasis and disease. Nat Rev Immunol (2017) 17:306–21. doi: 10.1038/nri.2017.11 28317925

[9] Zannetti C Roblot G Charrier E Ainouze M Tout I Briat F. Characterization of the inflammasome in human kupffer cells in response to synthetic agonists and pathogens. J Immunol (2016) 197:356–67. doi: 10.4049/jimmunol.1502301 27226092

[10] Gieseck RL Wilson MS Wynn TA. Type 2 immunity in tissue repair and fibrosis. Nat Rev Immunol (2018) 18:62–76. doi: 10.1038/nri.2017.90 28853443

[11] Tsuchida T Friedman SL. Mechanisms of hepatic stellate cell activation. Nat Rev Gastroenterol Hepatol (2017) 14:397–411. doi: 10.1038/nrgastro.2017.38 28487545

[12] Matsuda M Seki E. Hepatic stellate cell-macrophage crosstalk in liver fibrosis and carcinogenesis. Semin Liver Dis (2020) 40:307–20. doi: 10.1055/s-0040-1708876 PMC7484001 32242330

[13] di Carlo SE Peduto L. The perivascular origin of pathological fibroblasts. J Clin Invest (2018) 128:54–63. doi: 10.1172/JCI93558 29293094 PMC5749494

[14] Kostallari E Shah VH. Pericytes in the liver. Adv Exp Med Biol (2019) 1122:153–67. doi: 10.1007/978-3-030-11093-2_9 PMC7137998 30937868

[15] Ezhilarasan D. Hepatic stellate cells in the injured liver: Perspectives beyond hepatic fibrosis. J Cell Physiol (2022) 237:436–49. doi: 10.1002/jcp.30582 34514599

[16] Fabregat I Caballero-Díaz D. Transforming growth factor-β-induced cell plasticity in liver fibrosis and hepatocarcinogenesis. Front Oncol (2018) 8:357. doi: 10.3389/fonc.2018.00357 30250825 PMC6139328

[17] Wen Y Lambrecht J Ju C Tacke F. Hepatic macrophages in liver homeostasis and diseases-diversity, plasticity and therapeutic opportunities. Cell Mol Immunol (2021) 18:45–56. doi: 10.1038/s41423-020-00558-8 PMC7852525 33041338

[18] Kazlauskas A. PDGFs and their receptors. Gene (2017) 614:1–7. doi: 10.1016/j.gene.2017.03.003 28267575 PMC6728141

[19] Borkham-Kamphorst E Weiskirchen R. The PDGF system and its antagonists in liver fibrosis. Cytokine Growth Factor Rev (2016) 28:53–61. doi: 10.1016/j.cytogfr.2015.10.002 26547628

[20] Brito-Azevedo A Perez RM Maranhão PA Coelho HS Fernandes ESM Castiglione RC. Organ dysfunction in cirrhosis: A mechanism involving the microcirculation. Eur J Gastroenterol Hepatol (2019) 31:618–25. doi: 10.1097/MEG.0000000000001366 30920976

[21] Hintermann E Christen U. The many roles of cell adhesion molecules in hepatic fibrosis. Cells (2019) 8:1503. doi: 10.3390/cells8121503 PMC6952767 31771248

[22] Iwasaki A Sakai K Moriya K Sasaki T Keene DR Akhtar R. Molecular mechanism responsible for fibronectin-controlled alterations in matrix stiffness in advanced chronic liver fibrogenesis. J Biol Chem (2016) 291:72–88. doi: 10.1074/jbc.M115.691519 PMC4697189 26553870

[23] Jun J-I Lau LF. Resolution of organ fibrosis. J Clin Invest (2018) 128:97–107. doi: 10.1172/JCI93563 29293097 PMC5749507

[24] Kisseleva T Brenner D. Molecular and cellular mechanisms of liver fibrosis and its regression. Nat Rev Gastroenterol Hepatol (2021) 18:151–66. doi: 10.1038/s41575-020-00372-7 33128017

[25] Heymann F Tacke F. Immunology in the liver–from homeostasis to disease. Nat Rev Gastroenterol Hepatol (2016) 13:88–110. doi: 10.1038/nrgastro.2015.200 26758786

[26] Weston CJ Zimmermann HW Adams DH. The role of myeloid-derived cells in the progression of liver disease. Front Immunol (2019) 10:893. doi: 10.3389/fimmu.2019.00893 31068952 PMC6491757

[27] Guillot A Tacke F. Liver macrophages: Old dogmas and new insights. Hepatol Commun (2019) 3:730–43. doi: 10.1002/hep4.1356 PMC6545867 31168508

[28] Guilliams M Bonnardel J Haest B Vanderborght B Wagner C Remmerie A. Spatial proteogenomics reveals distinct and evolutionarily conserved hepatic macrophage niches. Cell (2022) 185:379–396.e38. doi: 10.1016/j.cell.2021.12.018 35021063 PMC8809252

[29] Elchaninov AV Fatkhudinov TK Vishnyakova PA Lokhonina AV Sukhikh GT. Phenotypical and functional polymorphism of liver resident macrophages. Cells (2019) 8:1032. doi: 10.3390/cells8091032 PMC6769646 31491903

[30] Scott CL Guilliams M. The role of kupffer cells in hepatic iron and lipid metabolism. J Hepatol (2018) 69:1197–9. doi: 10.1016/j.jhep.2018.02.013 PMC7611037 30001821

[31] Ginhoux F Guilliams M. Tissue-resident macrophage ontogeny and homeostasis. Immunity (2016) 44:439–49. doi: 10.1016/j.immuni.2016.02.024 26982352

[32] MacParland SA Liu JC Ma X-Z Innes BT Bartczak AM Gage BK. Single cell RNA sequencing of human liver reveals distinct intrahepatic macrophage populations. Nat Commun (2018) 9:4383. doi: 10.1038/s41467-018-06318-7 30348985 PMC6197289

[33] de Simone G Andreata F Bleriot C Fumagalli V Laura C Garcia-Manteiga JM. Identification of a kupffer cell subset capable of reverting the T cell dysfunction induced by hepatocellular priming. Immunity (2021) 54:2089–100.e8. doi: 10.1016/j.immuni.2021.05.005 34469774 PMC8459394

[34] Blériot C Barreby E Dunsmore G Ballaire R Chakarov S Ficht X. A subset of kupffer cells regulates metabolism through the expression of CD36. Immunity (2021) 54:2101–16.e6. doi: 10.1016/j.immuni.2021.08.006 34469775

[35] Soysa R Lampert S Yuen S Douglass AN Li W Pfeffer K. Fetal origin confers radioresistance on liver macrophages *via* p21cip1/WAF1. J Hepatol (2019) 71:553–62. doi: 10.1016/j.jhep.2019.04.015 PMC12509797 31077791

[36] Xiong X Kuang H Ansari S Liu T Gong J Wang S. Landscape of intercellular crosstalk in healthy and NASH liver revealed by single-cell secretome gene analysis. Mol Cell (2019) 75:644–60.e5. doi: 10.1016/j.molcel.2019.07.028 31398325 PMC7262680

[37] Fogg DK Sibon C Miled C Jung S Aucouturier P Littman DR. A clonogenic bone marrow progenitor specific for macrophages and dendritic cells. Science (2006) 311:83–7. doi: 10.1126/science.1117729 16322423

[38] Remmerie A Martens L Thoné T Castoldi A Seurinck R Pavie B. Osteopontin expression identifies a subset of recruited macrophages distinct from kupffer cells in the fatty liver. Immunity (2020) 53:641–57.e14. doi: 10.1016/j.immuni.2020.08.004 32888418 PMC7501731

[39] Sierro F Evrard M Rizzetto S Melino M Mitchell AJ Florido M. A liver capsular network of monocyte-derived macrophages restricts hepatic dissemination of intraperitoneal bacteria by neutrophil recruitment. Immunity (2017) 47:374–88.e6. doi: 10.1016/j.immuni.2017.07.018 28813662

[40] Cheng D Chai J Wang H Fu L Peng S Ni X. Hepatic macrophages: Key players in the development and progression of liver fibrosis. Liver Int (2021) 41:2279–94. doi: 10.1111/liv.14940 33966318

[41] Wang J Kubes P. A reservoir of mature cavity macrophages that can rapidly invade visceral organs to affect tissue repair. Cell (2016) 165:668–78. doi: 10.1016/j.cell.2016.03.009 27062926

[42] Mandal M Gardner CR Sun R Choi H Lad S Mishin V. The spleen as an extramedullary source of inflammatory cells responding to acetaminophen-induced liver injury. Toxicol Appl Pharmacol (2016) 304:110–20. doi: 10.1016/j.taap.2016.04.019 PMC5147741 27163765

[43] Bonnardel J T’Jonck W Gaublomme D Browaeys R Scott CL Martens L. Stellate cells, hepatocytes, and endothelial cells imprint the kupffer cell identity on monocytes colonizing the liver macrophage niche. Immunity (2019) 51:638–54.e9. doi: 10.1016/j.immuni.2019.08.017 31561945 PMC6876284

[44] Sakai M Troutman TD Seidman JS Ouyang Z Spann NJ Abe Y. Liver-derived signals sequentially reprogram myeloid enhancers to initiate and maintain kupffer cell identity. Immunity (2019) 51:655–70.e8. doi: 10.1016/j.immuni.2019.09.002 31587991 PMC6800814

[45] Wang C Ma C Gong L Guo Y Fu K Zhang Y. Macrophage polarization and its role in liver disease. Front Immunol (2021) 12:803037. doi: 10.3389/fimmu.2021.803037 34970275 PMC8712501

[46] Ritz T Krenkel O Tacke F. Dynamic plasticity of macrophage functions in diseased liver. Cell Immunol (2018) 330:175–82. doi: 10.1016/j.cellimm.2017.12.007 29454647

[47] Mohanty SK Donnelly B Temple H Ortiz-Perez A Mowery S Lobeck I. High mobility group box 1 release by cholangiocytes governs biliary atresia pathogenesis and correlates with increases in afflicted infants. Hepatology (2021) 74:864–78. doi: 10.1002/hep.31745 PMC8349381 33559243

[48] Gaskell H Ge X Nieto N. High-mobility group box-1 and liver disease. Hepatol Commun (2018) 2:1005–20. doi: 10.1002/hep4.1223 PMC6128227 30202816

[49] Singanayagam A Triantafyllou E. Macrophages in chronic liver failure: Diversity, plasticity and therapeutic targeting. Front Immunol (2021) 12:661182. doi: 10.3389/fimmu.2021.661182 33868313 PMC8051585

[50] Ramachandran P Pellicoro A Vernon MA Boulter L Aucott RL Ali A. Differential ly-6C expression identifies the recruited macrophage phenotype, which orchestrates the regression of murine liver fibrosis. Proc Natl Acad Sci U.S.A. (2012) 109:E3186–95. doi: 10.1073/pnas.1119964109 PMC3503234 23100531

[51] Zigmond E Samia-Grinberg S Pasmanik-Chor M Brazowski E Shibolet O Halpern Z. Infiltrating monocyte-derived macrophages and resident kupffer cells display different ontogeny and functions in acute liver injury. J Immunol (2014) 193:344–53. doi: 10.4049/jimmunol.1400574 24890723

[52] Ju C Tacke F. Hepatic macrophages in homeostasis and liver diseases: from pathogenesis to novel therapeutic strategies. Cell Mol Immunol (2016) 13:316–27. doi: 10.1038/cmi.2015.104 PMC4856798 26908374

[53] Dal-Secco D Wang J Zeng Z Kolaczkowska E Wong CHY Petri B. A dynamic spectrum of monocytes arising from the *in situ* reprogramming of CCR2+ monocytes at a site of sterile injury. J Exp Med (2015) 212:447–56. doi: 10.1084/jem.20141539 PMC4387291 25800956

[54] Gao J Wei B Liu M Hirsova P Sehrawat TS Cao S. Endothelial p300 promotes portal hypertension and hepatic fibrosis through c-c motif chemokine ligand 2–mediated angiocrine signaling. Hepatology (2021) 73:2468–83. doi: 10.1002/hep.31617 PMC8102654 33159815

[55] Ehling J Bartneck M Wei X Gremse F Fech V Möckel D. CCL2-dependent infiltrating macrophages promote angiogenesis in progressive liver fibrosis. Gut (2014) 63:1960–71. doi: 10.1136/gutjnl-2013-306294 PMC4216733 24561613

[56] Baeck C Wehr A Karlmark KR Heymann F Vucur M Gassler N. Pharmacological inhibition of the chemokine CCL2 (MCP-1) diminishes liver macrophage infiltration and steatohepatitis in chronic hepatic injury. Gut (2012) 61:416–26. doi: 10.1136/gutjnl-2011-300304 21813474

[57] Tacke F Weiskirchen R. An update on the recent advances in antifibrotic therapy. Expert Rev Gastroenterol Hepatol (2018) 12:1143–52. doi: 10.1080/17474124.2018.1530110 30261763

[58] Pradere J-P Kluwe J de Minicis S Jiao J-J Gwak G-Y Dapito DH. Hepatic macrophages but not dendritic cells contribute to liver fibrosis by promoting the survival of activated hepatic stellate cells in mice. Hepatology (2013) 58:1461–73. doi: 10.1002/hep.26429 PMC3848418 23553591

[59] Roh YS Seki E. Toll-like receptors in alcoholic liver disease, non-alcoholic steatohepatitis and carcinogenesis. J Gastroenterol Hepatol (Australia) (2013) 28:38–42. doi: 10.1111/jgh.12019 PMC3721430 23855294

[60] Karlmark KR Zimmermann HW Roderburg C Gassler N Wasmuth HE Luedde T. The fractalkine receptor CX₃CR1 protects against liver fibrosis by controlling differentiation and survival of infiltrating hepatic monocytes. Hepatology (2010) 52:1769–82. doi: 10.1002/hep.23894 21038415

[61] Aoyama T Inokuchi S Brenner DA Seki E. CX3CL1-CX3CR1 interaction prevents carbon tetrachloride-induced liver inflammation and fibrosis in mice. Hepatology (2010) 52:1390–400. doi: 10.1002/hep.23795 PMC2947579 20683935

[62] Roohani S Tacke F. Liver injury and the macrophage issue: Molecular and mechanistic facts and their clinical relevance. Int J Mol Sci (2021) 22:7249.. doi: 10.3390/ijms22147249 34298870 PMC8306699

[63] Ramachandran P Dobie R Wilson-Kanamori JR Dora EF Henderson BEP Luu NT. Resolving the fibrotic niche of human liver cirrhosis at single-cell level. Nature (2019) 575:512–8. doi: 10.1038/s41586-019-1631-3 PMC6876711 31597160

[64] Seki E Schwabe RF. Hepatic inflammation and fibrosis: functional links and key pathways. Hepatology (2015) 61:1066–79. doi: 10.1002/hep.27332 PMC4306641 25066777

[65] Affò S Morales-Ibanez O Rodrigo-Torres D Altamirano J Blaya D Dapito DH. CCL20 mediates lipopolysaccharide induced liver injury and is a potential driver of inflammation and fibrosis in alcoholic hepatitis. Gut (2014) 63:1782–92. doi: 10.1136/gutjnl-2013-306098 PMC4092046 24415562

[66] Inokuchi S Tsukamoto H Park E Liu Z-X Brenner DA Seki E. Toll-like receptor 4 mediates alcohol-induced steatohepatitis through bone marrow-derived and endogenous liver cells in mice. Alcohol Clin Exp Res (2011) 35:1509–18. doi: 10.1111/j.1530-0277.2011.01487.x PMC3131439 21463341

[67] Zhao Q Fan Y-C Zhao J Gao S Zhao Z-H Wang K. DNA Methylation patterns of peroxisome proliferator-activated receptor gamma gene associated with liver fibrosis and inflammation in chronic hepatitis b. J Viral Hepat (2013) 20:430–7. doi: 10.1111/jvh.12048 23647960

[68] Mann J Chu DCK Maxwell A Oakley F Zhu N-L Tsukamoto H. MeCP2 controls an epigenetic pathway that promotes myofibroblast transdifferentiation and fibrosis. Gastroenterology (2010) 138:705–14. doi: 10.1053/j.gastro.2009.10.002 PMC2819585 19843474

[69] Lefere S Puengel T Hundertmark J Penners C Frank AK Guillot A. Differential effects of selective- and pan-PPAR agonists on experimental steatohepatitis and hepatic macrophages☆. J Hepatol (2020) 73:757–70. doi: 10.1016/j.jhep.2020.04.025 32360434

[70] Zhang K Shi Z Zhang M Dong X Zheng L Li G. Silencing lncRNA Lfar1 alleviates the classical activation and pyoptosis of macrophage in hepatic fibrosis. Cell Death Dis (2020) 11:132. doi: 10.1038/s41419-020-2323-5 32071306 PMC7028920

[71] Jeong W-I Park O Suh Y-G Byun J-S Park S-Y Choi E. Suppression of innate immunity (natural killer cell/interferon-γ) in the advanced stages of liver fibrosis in mice. Hepatology (2011) 53:1342–51. doi: 10.1002/hep.24190 PMC3079530 21480338

[72] Read SA Wijaya R Ramezani-Moghadam M Tay E Schibeci S Liddle C. Macrophage coordination of the interferon lambda immune response. Front Immunol (2019) 10:2674. doi: 10.3389/fimmu.2019.02674 31798594 PMC6878940

[73] Inagaki Y Nemoto T Kushida M Sheng Y Higashi K Ikeda K. Interferon alfa down-regulates collagen gene transcription and suppresses experimental hepatic fibrosis in mice. Hepatology (2003) 38:890–9. doi: 10.1053/jhep.2003.50408 14512876

[74] Seki E Brenner DA Karin M. A liver full of JNK: signaling in regulation of cell function and disease pathogenesis, and clinical approaches. Gastroenterology (2012) 143:307–20. doi: 10.1053/j.gastro.2012.06.004 PMC3523093 22705006

[75] Gautheron J Vucur M Reisinger F Cardenas DV Roderburg C Koppe C. A positive feedback loop between RIP 3 and JNK controls non-alcoholic steatohepatitis. EMBO Mol Med (2014) 6:1062–74. doi: 10.15252/emmm.201403856 PMC4154133 24963148

[76] Kluwe J Pradere J-P Gwak G-Y Mencin A de Minicis S Osterreicher CH. Modulation of hepatic fibrosis by c-Jun-N-terminal kinase inhibition. Gastroenterology (2010) 138:347–59. doi: 10.1053/j.gastro.2009.09.015 PMC2988578 19782079

[77] Son G Iimuro Y Seki E Hirano T Kaneda Y Fujimoto J. Selective inactivation of NF-kappaB in the liver using NF-kappaB decoy suppresses CCl4-induced liver injury and fibrosis. Am J Physiol Gastrointest Liver Physiol (2007) 293:G631–9. doi: 10.1152/ajpgi.00185.2007 17640975

[78] Zhao J Qi Y-F Yu Y-R. STAT3: A key regulator in liver fibrosis. Ann Hepatol (2021) 21:100224. doi: 10.1016/j.aohep.2020.06.010 32702499

[79] Bharadwaj U Kasembeli MM Robinson P Tweardy DJ. Targeting janus kinases and signal transducer and activator of transcription 3 to treat inflammation, fibrosis, and cancer: Rationale, progress, and caution. Pharmacol Rev (2020) 72:486–526. doi: 10.1124/pr.119.018440 32198236 PMC7300325

[80] He F Guo F-C Li Z Yu H-C Ma P-F Zhao J-L. Myeloid-specific disruption of recombination signal binding protein jκ ameliorates hepatic fibrosis by attenuating inflammation through cylindromatosis in mice. Hepatology (2015) 61:303–14. doi: 10.1002/hep.27394 25145286

[81] Zheng S Zhang P Chen Y Zheng S Zheng L Weng Z. Inhibition of notch signaling attenuates schistosomiasis hepatic fibrosis *via* blocking macrophage M2 polarization. PLoS One (2016) 11:e0166808. doi: 10.1371/journal.pone.0166808 27875565 PMC5119780

[82] Bansal R van Baarlen J Storm G Prakash J. The interplay of the notch signaling in hepatic stellate cells and macrophages determines the fate of liver fibrogenesis. Sci Rep (2015) 5:18272. doi: 10.1038/srep18272 26658360 PMC4677309

[83] Ye YC Zhao JL Lu YT Gao CC Yang Y Liang SQ. Notch signaling *via* wnt regulates the proliferation of alternative, CCR2-independent tumor-associated macrophages in hepatocellular carcinoma. Cancer Res (2019) 79:4160–72. doi: 10.1158/0008-5472.CAN-18-1691 31266773

[84] Distler JHW Györfi A-H Ramanujam M Whitfield ML Königshoff M Lafyatis R. Shared and distinct mechanisms of fibrosis. Nat Rev Rheumatol (2019) 15:705–30. doi: 10.1038/s41584-019-0322-7 31712723

[85] Tian L Wang Y Jang YY. Wnt signaling in biliary development, proliferation, and fibrosis. Exp Biol Med (Maywood) (2022) 247:360–7. doi: 10.1177/15353702211061376 PMC8899336 34861115

[86] Liedtke C Luedde T Sauerbruch T Scholten D Streetz K Tacke F. Experimental liver fibrosis research: update on animal models, legal issues and translational aspects. Fibrogene Tissue Repair (2013) 6:19. doi: 10.1186/1755-1536-6-19 PMC3850878 24274743

[87] Schlitzer A Schultze JL. Tissue-resident macrophages - how to humanize our knowledge. Immunol Cell Biol (2017) 95:173–7. doi: 10.1038/icb.2016.82 27752049

[88] Tacke F. Targeting hepatic macrophages to treat liver diseases. J Hepatol (2017) 66:1300–12. doi: 10.1016/j.jhep.2017.02.026 28267621

[89] Seki E de Minicis S Osterreicher CH Kluwe J Osawa Y Brenner DA. TLR4 enhances TGF-beta signaling and hepatic fibrosis. Nat Med (2007) 13:1324–32. doi: 10.1038/nm1663 17952090

[90] Loomba R Lawitz E Mantry PS Jayakumar S Caldwell SH Arnold H. The ASK1 inhibitor selonsertib in patients with nonalcoholic steatohepatitis: A randomized, phase 2 trial. Hepatology (2018) 67:549–59. doi: 10.1002/hep.29514 PMC5814892 28892558

[91] Dong X Liu J Xu Y Cao H. Role of macrophages in experimental liver injury and repair in mice (Review). Exp Ther Med (2019) 17:3835–47. doi: 10.3892/etm.2019.7450 PMC6468932 31007731

[92] Lefebvre E Moyle G Reshef R Richman LP Thompson M Hong F. Antifibrotic effects of the dual CCR2/CCR5 antagonist cenicriviroc in animal models of liver and kidney fibrosis. PloS One (2016) 11:e0158156. doi: 10.1371/journal.pone.0158156 27347680 PMC4922569

[93] Mossanen JC Krenkel O Ergen C Govaere O Liepelt A Puengel T. Chemokine (C-c motif) receptor 2-positive monocytes aggravate the early phase of acetaminophen-induced acute liver injury. Hepatology (2016) 64:1667–82. doi: 10.1002/hep.28682 27302828

[94] Jalan R Fernandez J Wiest R Schnabl B Moreau R Angeli P. Bacterial infections in cirrhosis: a position statement based on the EASL special conference 2013. J Hepatol (2014) 60:1310–24. doi: 10.1016/j.jhep.2014.01.024 24530646

[95] Stutchfield BM Antoine DJ Mackinnon AC Gow DJ Bain CC Hawley CA. CSF1 restores innate immunity after liver injury in mice and serum levels indicate outcomes of patients with acute liver failure. Gastroenterology (2015) 149:1896–909.e14. doi: 10.1053/j.gastro.2015.08.053 26344055 PMC4672154

[96] Ergen C Heymann F Al Rawashdeh W Gremse F Bartneck M Panzer U. Targeting distinct myeloid cell populations in vivo using polymers, liposomes and microbubbles. Biomater (2017) 114:106–20. doi: 10.1016/j.biomaterials.2016.11.009 27855336

[97] Bartneck M Scheyda KM Warzecha KT Rizzo LY Hittatiya K Luedde T. Fluorescent cell-traceable dexamethasone-loaded liposomes for the treatment of inflammatory liver diseases. Biomaterials (2015) 37:367–82. doi: 10.1016/j.biomaterials.2014.10.030 25453965

[98] Bartneck M Warzecha KT Tacke F. Therapeutic targeting of liver inflammation and fibrosis by nanomedicine. Hepatobil Surg Nutr (2014) 3:364–76. doi: 10.3978/j.issn.2304-3881.2014.11.02 PMC4273112 25568860

[99] Traber PG Zomer E. Therapy of experimental NASH and fibrosis with galectin inhibitors. PloS One (2013) 8:e83481. doi: 10.1371/journal.pone.0083481 24367597 PMC3867460

[100] Moore JK Mackinnon AC Wojtacha D Pope C Fraser AR Burgoyne P. Phenotypic and functional characterization of macrophages with therapeutic potential generated from human cirrhotic monocytes in a cohort study. Cytotherapy (2015) 17:1604–16. doi: 10.1016/j.jcyt.2015.07.016 PMC4596388 26342993

[101] Moroni F Dwyer BJ Graham C Pass C Bailey L Ritchie L. Safety profile of autologous macrophage therapy for liver cirrhosis. Nat Med (2019) 25:1560–5. doi: 10.1038/s41591-019-0599-8 31591593

[102] Thomas JA Pope C Wojtacha D Robson AJ Gordon-Walker TT Hartland S. Macrophage therapy for murine liver fibrosis recruits host effector cells improving fibrosis, regeneration, and function. Hepatology (2011) 53:2003–15. doi: 10.1002/hep.24315 21433043

[103] Ma P-F Gao C-C Yi J Zhao J-L Liang S-Q Zhao Y. Cytotherapy with M1-polarized macrophages ameliorates liver fibrosis by modulating immune microenvironment in mice. J Hepatol (2017) 67:770–9. doi: 10.1016/j.jhep.2017.05.022 28596109

[104] Ma P Han H Qin H. Reply to: “Studies of macrophage therapy for cirrhosis - from mice to men”. J Hepatol (2018) 68:1091–3. doi: 10.1016/j.jhep.2017.12.024 29317296

[105] Poltavets AS Vishnyakova PA Elchaninov AV Sukhikh GT Fatkhudinov TK. Macrophage modification strategies for efficient cell therapy. Cells (2020) 9:1535. doi: 10.3390/cells9061535 PMC7348902 32599709

[106] Essandoh K Li Y Huo J Fan G-C. MiRNA-mediated macrophage polarization and its potential role in the regulation of inflammatory response. Shock (2016) 46:122–31. doi: 10.1097/SHK.0000000000000604 PMC4949115 26954942

[107] Zhou J-E Sun L Liu L Jia Y Han Y Shao J. Hepatic macrophage targeted siRNA lipid nanoparticles treat non-alcoholic steatohepatitis. J Control Release (2022) 343:175–86. doi: 10.1016/j.jconrel.2022.01.038 35092721

